# Reactive Oxygen Species Are Essential for Vasoconstriction upon Cold Exposure

**DOI:** 10.1155/2021/8578452

**Published:** 2021-11-24

**Authors:** Di Zhang, Shiquan Chang, Bei Jing, Xin Li, Huimei Shi, Yachun Zheng, Yi Lin, Zhenni Chen, Guoqiang Qian, Yuwei Pan, Guoping Zhao

**Affiliations:** ^1^College of Traditional Chinese Medicine, Jinan University, Guangzhou, China; ^2^Guangdong Pharmaceutical University, Guangzhou, China; ^3^Tianhe Hospital of Traditional Chinese Medicine, Guangzhou, China

## Abstract

**Purpose:**

We explored the role of ROS in cold-induced vasoconstriction and corresponding mechanism.

**Methods:**

Three experiments were performed. First, we measured blood flow in human hands before and after cold exposure. Second, 24 mice were randomly divided into 3 groups: 8 mice received saline injection, 8 received subcutaneous Tempol injection, and 8 received intrathecal Tempol injection. After 30 min, we determined blood flow in the skin before and after cold exposure. Finally, we used Tempol, CCG-1423, and Go 6983 to pretreat HAVSMCs and HUVECs for 24 h. Then, cells in the corresponding groups were exposed to cold (6 h, 4°C). After cold exposure, the cytoskeleton was stained. Intracellular Ca^2+^ and ROS levels were measured by flow cytometry and fluorescence microscopy. We measured protein expression via Western blotting.

**Results:**

In the first experiment, after cold exposure, maximum skin blood flow decreased to 118.4 ± 50.97 flux units. Then, Tempol or normal saline pretreatment did not change skin blood flow. Unlike intrathecal Tempol injection, subcutaneous Tempol injection increased skin blood flow after cold exposure. Finally, cold exposure for 6 h shrank the cells, making them narrower, and increased intracellular Ca^2+^ and ROS levels in HUVECs and HAVSMCs. Tempol reduced cell shrinkage and decreased intracellular Ca^2+^ levels. In addition, Tempol decreased intracellular ROS levels. Cold exposure increased RhoA, Rock1, p-MLC-2, ET-1, iNOS, and p-PKC expression and decreased eNOS expression. Tempol or CCG-1423 pretreatment decreased RhoA, Rock1, and p-MLC-2 levels in HAVSMCs. Furthermore, Tempol or Go 6983 pretreatment decreased ET-1, iNOS, and p-PKC expression and increased eNOS expression in HUVECs.

**Conclusion:**

ROS mediate the vasoconstrictor response within the cold-induced vascular response, and ROS in blood vessel tissues rather than nerve fibers are involved in vasoconstriction via the ROS/RhoA/ROCK1 and ROS/PKC/ET-1 pathways in VSMCs and endothelial cells.

## 1. Introduction

With global climate extremes increasing, ambient temperature has become extensively important for various health outcomes and increasingly attracted research attention [1]. A large epidemiological studies have shown that cold exposure increases the morbidity and mortality of cardiovascular and respiratory diseases [2–4]. Cold exposure in mammals results in rapid-onset vasoconstriction to protect against heat loss [5]. The mechanisms involved in this vascular response to cold have been under study for decades.

Vasoconstriction in a cold environment is regulated by nerves, vascular smooth muscle, and endothelial cells. Mechanistically, cold-induced vasoconstriction results from a reflex reaction mediated by neuronal (norepinephrine) and local effectors that increase vascular sensitization to cold [6]. Sympathetic noradrenergic vasoconstrictor nerves cause a rapid decrease in skin blood flow, thus increasing the insulative capacity of the skin and decreasing heat loss from the body. Although neuromodulation is vital to this process, sympathetic nonnoradrenergic cotransmitter(s) contribute to reflex vasoconstriction [7]. In addition to neuromodulation, nonneuromodulatory mechanisms play a critical role in vasoconstriction. ROS played a vital role in vasoconstriction after cold exposure. Pan et al. [8] found that increasing endothelial nitric oxide synthase (eNOS) activation and NO release and inhibiting NADPH oxidase-derived ROS generation could improve vascular function, preventing the development and progression of hypertension vasculopathy. However, it is not clear how ROS regulate vasoconstriction or where ROS exert their effects.

RhoA/ROCK pathway activation played a vital role in coronary artery spasm [9]. Besides, inhibition of RhoA/ROCK signaling pathway ameliorates hypoxic pulmonary hypertension [10]. PKC inhibitor can significantly inhibit potentiation of capsaicin-induced constriction by endothelin-1 [11], which indicates that PKC is involved in vasoconstriction. Thus, in this study, we determined that extraneural ROS are mainly involved in vasoconstriction via the ROS/RhoA/ROCK1 and ROS/PKC/ET-1 pathways in vascular smooth muscle cells (VSMCs) and endothelial cells.

## 2. Methods

### 2.1. Participants

Experiments in which hands were subjected to cold exposure were approved by the Ethics Committee of Tianhe Hospital of Traditional Chinese Medicine of Jinan University. All the participants were fully informed of the methods and risks before informed consent was obtained. A total of 4 male and 3 female participants took part in this study (age: 25 ± 3 years; height: 165 ± 10 cm; weight: 56 ± 5 kg). All the participants were healthy nonsmokers; did not take medications; and were free of cardiovascular, neurological, and metabolic diseases. All refrained from alcoholic and caffeinated beverages for at least 12 h prior to the study. All the studies were performed over a one-week period in December 2020 in Guangdong.

### 2.2. Mice and Cell Lines

Twenty-four male ICR mice (8–12 weeks of age) were acquired from Beijing Vital River Laboratories. All mice were allowed to acclimate for 10 days before we used depilatory cream to remove the hair from each mouse's lower limbs and buttocks. Then, we continued to feed the mice for a week. Animal experiments were approved by the Experimental Animal Ethics Committee of Jinan University (ethics number: 20210104-01). Aloe vera gel was applied to the skin for a week after hair removal to promote skin return to normal. T/G HA-VSMCs were purchased from Shenzhen Kuyuan Biotechnology Co., Ltd. (Guangzhou, China). Human umbilical vein endothelial cells (HUVECs) were purchased from Procell Life Science & Technology Co., Ltd. (Wuhan, China).

### 2.3. Reagents

A Cell Counting Kit-8 (96992) was obtained from Guangzhou Juyan Biological Co., Ltd. (Guangzhou, China). PageR*μ*Ler Prestained Protein Ladder and Marker (AG11919) was obtained from Accurate Biotechnology Co., Ltd. (Changsha, China). A phosphatase inhibitor cocktail (G2007) and a BCA protein content kit (CW0014S) were acquired from Guangzhou SIJIA Biotechnology Co., Ltd. (Guangzhou, China). Tempol (an active oxygen neutralizer, T6699), CCG-1423 (a RhoA inhibitor, T2014), and Go 6983 (a PKC inhibitor, M2019) were acquired from Guangzhou Yiyou Biotechnology Biological Co., Ltd. (Guangzhou, China). Rabbit anti-p-PKC (2060S), anti-*β*-actin (4970S), anti-eNOS (9586S), anti-iNOS (2982S), and anti-p-MLC-2 (3675S) were acquired from Cell Signaling Technology (Boston, USA). Rabbit anti-RhoA (A5651), anti-ROCK1 (A5141), anti-ROCK2 (A5156), anti-MLC-2 (A5655), anti-endothelin 1 (ET-1) (A0686), and anti-PKC (pan) (A17921) were purchased from Selleck Biotechnology Biological Co., Ltd. (Shanghai, China). We purchased DCFH-DA (D6883), Fluo3-AM (F809977), and a cytoskeleton staining kit (BB-4440) from Guangzhou Junji Biotechnology Co., Ltd. (Guangzhou, China).

### 2.4. Measurement of Cutaneous Blood Flow in the Hands

We utilized a laser speckle blood flow imaging system (Moor Instruments, UK) to scan the surface of the back of each volunteer's right hand for 100 s. Then, the hands were placed in cold water at 4°C for 5 minutes and dried with paper towels. Then, we continued to monitor hand blood flow for 200 s. The distance between the scan head and hand surface was approximately 20 cm. The image acquisition rate was one frame per second, and images were acquired in normal resolution mode.

### 2.5. Scanning of the Microblood Flow on the Mouse Hind Limb Surface

After 12 h of fasting, 24 mice were randomly divided into 3 groups: 8 mice were injected with saline (control group), 8 mice were subcutaneously injected with Tempol (30 mg/kg, dissolved in saline, Tempol subcutaneous injection group), and 8 mice received Tempol by intrathecal injection (20 *μ*g/kg, dissolved in saline, Tempol intrathecal injection group). Then, all mice were anesthetized with an intraperitoneal injection of 2% pentobarbital (8 mg/100 g body weight). Thirty minutes after injection, we scanned changes in blood flow in the buttocks and limbs for 120 s. Then, we placed the lower limbs and buttocks in cold water at 4°C for 5 minutes and dried these parts with paper towels. Last, we scanned the lower limbs and buttocks to detect blood flow for 180 s with the laser speckle blood flow imaging system using the same parameters used for the previous experiment.

### 2.6. Cell Culture

Human VSMCs (HAVSMCs) and HUVECs were cultured with 10% FBS+89% DMEM+1% antibiotics at the Formula-Pattern Research Center (School of Traditional Chinese Medicine, Jinan University). The HAVSMCs and HUVECs were seeded in 96-well plates at a density of 6 × 10^3^ cells/well. We examined the effects of Tempol, CCG-1423, and Go 6983 by CCK-8 assay, and we chose the concentrations of 10 *μ*M, 10 *μ*M, and 10 *μ*M, respectively. Cells between passages 6 and 12 were used for the experiments.

### 2.7. Grouping and Drug Concentrations Used for Cellular Experiments

We pretreated HAVSMCs and HUVECs with Tempol, CCG-1423, and Go 6983 for 24 h. Then, we exchanged the medium, and the cells were subjected to cold exposure. Thus, for HAVSMC experiments, four groups were used: the control group (37°C for 6 h), model group (4°C cold exposure for 6 h), Tempol group (4°C cold exposure for 6 h +10 *μ*M Tempol), and CCG-1423 group (4°C cold exposure for 6 h +10 *μ*M CCG 1423). For HUVEC experiments, four groups were used: the control group (37°C for 6 h), model group (4°C cold exposure for 6 h), Tempol group (4°C cold exposure for 6 h +10 *μ*M Tempol), and Go 6983 group (4°C cold exposure for 6 h +10 *μ*M Go 6983).

### 2.8. Intracellular ROS Measurements

An intracellular ROS measurement assay was performed as previously described [12, 13]. After cold exposure, the cells were collected in EP tubes. The cells were then incubated for 20 min in PBS containing 20 *μ*M DCFH-DA in the dark. Then, the DCFH-DA was removed, and we used PBS to wash the cells 3 times. Finally, intracellular ROS production was measured on a flow cytometer. In addition, cells in a 6-well plate subjected to cold exposure were incubated for 20 min in PBS containing 20 *μ*M DCFH-DA in the dark. After these, we used PBS to wash the cells 3 times. Intracellular ROS production was measured on an inverted fluorescence microscope. We determined the extent of intracellular ROS produced based on the fluorescence intensity via Image-Pro Plus 6.0 software.

### 2.9. Detection of the Intracellular Ca^2+^ Concentration

After cold exposure, the cells were collected in EP tubes, and other cells were seeded in 6-well plates. All cells were incubated for 30 min in HBSS containing 1 *μ*M Fluo-3 AM in the dark. Then, the Fluo-3 AM was removed, and we used HBSS to wash the cells 3 times. Finally, the intracellular Ca^2+^ concentration was measured by flow cytometry and fluorescence microscopy.

### 2.10. Cytoskeleton Experiment

Cells were seeded on cell slides in a 6-well plate. According to the protocol, after the cells were washed, permeabilized, fixed, and stained, the cell morphology was observed using an optical microscope.

### 2.11. Western Blot Analysis

The levels of RhoA, ROCK1, ROCK2, Pan PKC, p-PKC, MLC-2, p-MLC-2, ET-1, iNOS, and eNOS were measured by Western blotting. RIPA buffer was used to lyse the cells and obtain the proteins from the supernatant. The protein concentration was determined via a BCA assay, and samples (30 *μ*g) were separated via 4-10% SDS-PAGE followed by transfer to PVDF membranes and blocking with 5% skim milk at 37°C for 1 h. After these, PVDF membranes were incubated with primary antibody (1 : 1000) overnight at 4°C for 12 h and incubated with secondary antibody (1 : 30000) for 1 h. Finally, chemiluminescence was used to visualize the blots.

### 2.12. Statistical Analyses

Cell fluorescence was analyzed by Image-Pro Plus 6.0 software. We analyzed the results of flow cytometry experiments using FlowJo v10. Values are expressed as the mean ± standard deviation. We conducted all statistical analyses and generated graphs with GraphPad Prism 8, Adobe Illustrator 2020 software. The data of continuously measured blood flow of mice were analyzed by repeated measure ANOVA. The other data were analyzed by one-way ANOVA. Tukey's multiple comparisons test was the post hoc test after ANOVA. *p* < 0.05 was considered to indicate a statistically significant difference.

## 3. Results

### 3.1. Cold Exposure Caused Vasoconstriction

**Figure 1:**
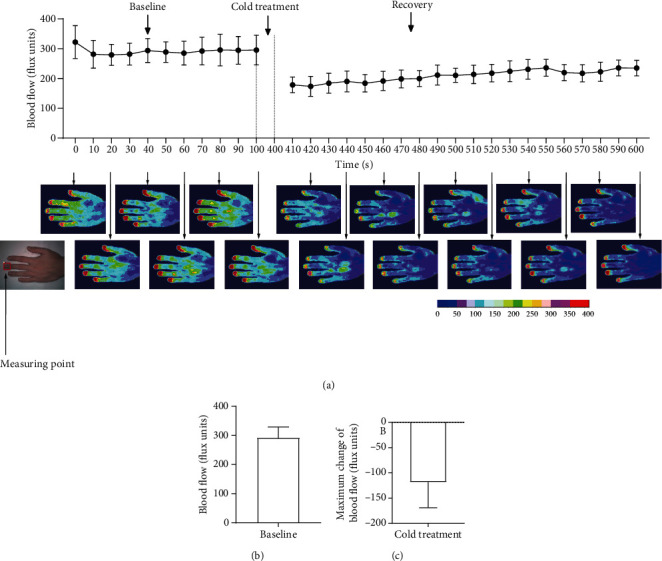
Cold exposure causes vasoconstriction. Cold exposure decreases blood flow to the skin. Cold exposure decreased blood flow to the fingertips by 118.4 flux units on average.

Following baseline blood flow measurements, the right hand was immersed in cold water (4°C for 5 min, Figure 1(a), *n* = 7). The maximum vasoconstriction was observed after 410 to 460 s. At room temperature, the blood flow at the tip of the finger was 291.9 ± 37.1 flux units (Figure 1(b)). After cold exposure, the maximum drop in blood flow was 118.4 ± 50.97 flux units at 420 s (Figure 1(c)). These results indicate that cold stimulation reduced skin blood flow.

### 3.2. Tempol Exposure Increased Blood Flow after Cold Exposure

**Figure 2:**
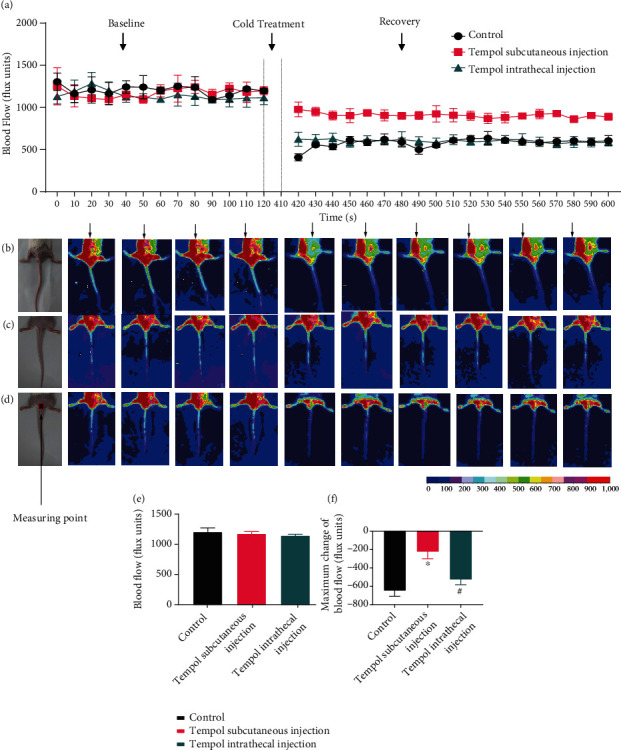
Tempol increased blood flow after cold exposure. Cold stimulation induced vasoconstriction and reduced blood flow of the skin. The reduction in ROS levels in blood vessels reversed this change, but reducing ROS levels in nerve fibers did not have this effect (*n* = 8, *p* < 0.05). ^∗^Compared with the control group. ^#^Compared with the Tempol subcutaneous injection group.

Following baseline blood flow measurements, the two lower limbs and buttocks were immersed in cold water (4°C for 5 min, Figure 2). After cold exposure, blood flow in the skin on the tail and buttocks decreased. Subcutaneous injection of Tempol significantly increased skin blood flow after cold stimulation, while intrathecal injection of Tempol did not significantly increase skin blood flow (Figures 2(a)–2(d)). As shown in Figure 2(e), there was no difference in baseline blood flow of the skin among the three groups. Vasoconstriction was greatest at 420 s to 470 s following local cooling and determined as the % maximum decrease in blood flow from precooling baseline blood flow (Figures 2(a)–2(f), *p* < 0.05). Given the fact that the control blood flow at 420 s is much smaller that for all following points in the absence of a visible recovery during the observation period from 430 s to 600 s (Figures 2(b)–2(d), *p* < 0.05), we consider this might be an artifact. Therefore, in the statistical process, we remove this point. After cold exposure, the drops in blood flow at 430 s were 648.5 ± 59.18, 223.9 ± 75.33, and 526.5 ± 56.70 flux units, respectively (Figure 2(f)). These responses indicate that ROS mediate the vasoconstrictor response in the cold-induced vascular response and that ROS in blood vessel tissues rather than nerve fibers are involved in vasoconstriction.

### 3.3. Cold Increased ROS Levels in HAVSMCs (Figure 3(a))

**Figure 3:**
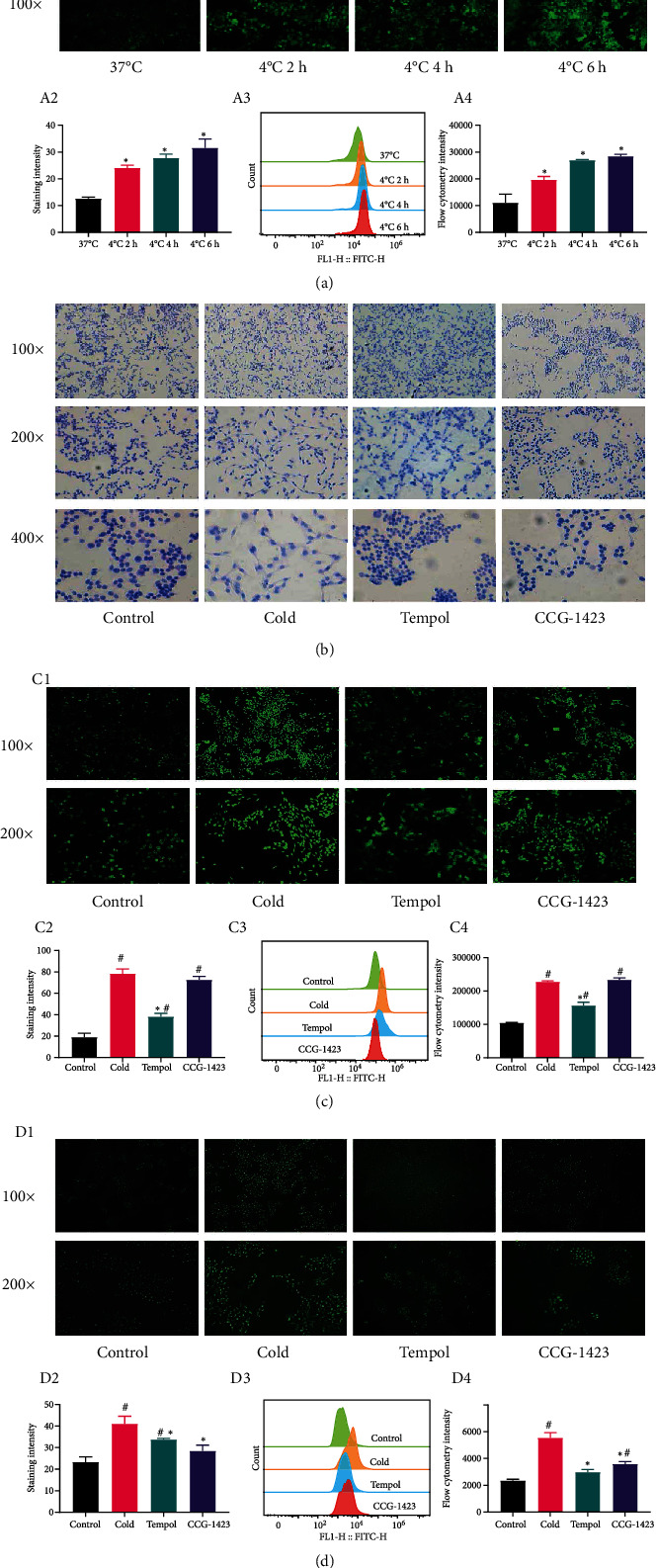
The impact of cold on HAVSMCs. (a) Cold stimulation for 6 h increased ROS levels (A1 shows ROS fluorescence, A3 shows flow cytometry data, and A2 and A4 show the results of statistical analysis; *p* < 0.05). (b) Cold caused the cells to shrink, while Tempol and CCG-1423 prevented cell shrinkage and restored cells to their baseline form. (c) Cold increased intracellular ROS levels, and Tempol, but not CCG-1423, reduced intracellular ROS levels (C1 shows ROS fluorescence, C3 shows flow cytometry data, and C2 and C4 show the results of statistical analysis; *p* < 0.05). (d) Cold increased intracellular Ca^2+^ levels, and Tempol and CCG-1423 prevented this this (D1 shows Ca^2+^ fluorescence, D3 shows flow cytometry data, and D2 and D4 show the results of statistical analysis; *p* < 0.05). The experiments were repeated three times. ^∗^Compared with the cold group. ^#^Compared with the control group.

To explore the relationship between the duration of cold exposure and increase in ROS, we examined ROS levels after cold exposure for different durations (2 h, 4 h, and 6 h) via flow cytometry and fluorescence microscopy (Figure 3(a)). As shown in Figure 3, (A1–A2), cold exposure for 6 h significantly increased ROS levels compared to those at 37°C (ROS fluorescence, *p* < 0.05). The data in Figure 3, (A3–A4) confirmed these results (ROS fluorescence, *p* < 0.05). Therefore, cold exposure for 6 h was suitable for follow-up studies.

### 3.4. Cold Exposure Changed the Shape of HAVSMCs (Figure 3(b))

We detected the shape of HAVSMCs by staining the cytoskeleton. As shown in Figure 3(b), HAVSMCs in the control group were fusiform and flat. After cold stimulation, we observed cell shrinkage and the presence of irregular edges, and the cells were flatter than those in the control group. Tempol and CCG-1423 treatment changed the cell morphology and inhibited cell shrinkage (Figure 3(b)).

### 3.5. Tempol Decreased Intracellular ROS Levels in HAVSMCs (Figure 3(c))

We examined intracellular ROS levels in the different groups of HAVSMCs after cold exposure for 6 h via fluorescence microscopy and flow cytometry (Figure 3(c)). As shown in Figure 3, (C1–C4), cold exposure increased intracellular ROS levels (*p* < 0.05), and Tempol treatment reduced ROS levels (*p* < 0.05), while CCG-1423 did not decrease ROS levels compared to those in the cold group (*p* < 0.05). The results of flow cytometry (Figure 3, C3–C4) were consistent with those of the fluorescence experiment (Figure 3, C1–C2). This experiment showed that a RhoA inhibitor (CCG-1423) did not affect intracellular ROS levels.

### 3.6. Tempol and CCG-1423 Decreased Intracellular Ca^2+^ Levels in HAVSMCs (Figure 3(d))

We detected the intracellular Ca^2+^ levels of the different groups of HAVSMCs after cold exposure through fluorescence microscopy and flow cytometry (Figure 3(d)). As shown in Figure 3, (D1–D2), cold exposure increased intracellular Ca^2+^ levels (*p* < 0.05), and Tempol and CCG-1423 abrogated this change (*p* < 0.05) compared to the cold group (*p* < 0.05). In addition, we used flow cytometry to detect the intracellular Ca^2+^ concentration. We found that cold exposure increased the Ca^2+^ concentration (*p* < 0.05), but Tempol and CCG-1423 abrogated this change (*p* < 0.05). Thus, the results of flow cytometry and fluorescence microscopy were consistent.

### 3.7. Expression of ROS/RhoA/ROCK1 Pathway-Related Proteins in HAVSMCs (Figure 4)

Cold led to changes in the expression of ROS/RhoA/ROCK1 pathway-related proteins in HAVSMCs. The expression levels of Rock2 and MLC-2 in the four groups were essentially the same. Cold increased the expression of RhoA, Rock1, and p-MLC-2 compared to those in the other groups (*p* < 0.05). Tempol and CCG-1423 decreased the levels of RhoA, Rock1, and p-MLC-2 compared to those in the cold group (*p* < 0.05).

### 3.8. Cold Led HUVECs to Shrink (Figure 5(a))

**Figure 5:**
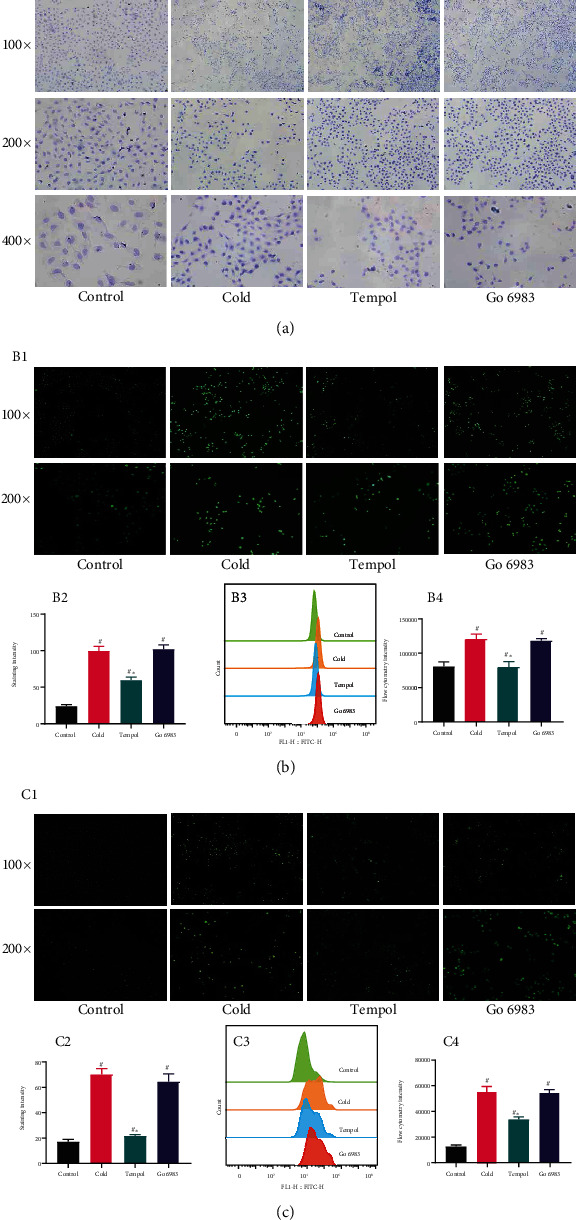
The impact of cold on HUVECs. (a) Cold caused cell shrinkage, and Tempol and CCG-1423 prevented this change, restoring cells to their baseline form. (b) Cold exposure increased intracellular ROS levels, and Tempol, but not Go 6983, reduced intracellular ROS levels (B1 shows ROS fluorescence, B3 shows flow cytometry data, and B2 and B4 show the results of statistical analysis; *p* < 0.05). (c) Cold increased intracellular Ca^2+^ levels, and Tempol but not Go 6983 prevented this increase (C1 shows ROS fluorescence, C3 shows flow cytometry data, and C2 and C4 show the results of statistical analysis; *p* < 0.05). The experiments were repeated three times. ^∗^Compared with the cold group. ^#^Compared with the control group.

As in previous experiments, the cells were still subjected to cold exposure for 6 h. As shown in Figure 5(a), cold exposure caused the cells to shrink and become thinner. Pretreatment with Tempol and Go 6983 reversed these changes and prevented the cells from shrinking.

### 3.9. Tempol Decreased Intracellular ROS Levels in HUVECs (Figure 5(b))

ROS fluorescence data and flow cytometry showed that cold exposure increased intracellular ROS levels (Figure 5, (B1 and B3), *p* < 0.05), consistent with the results of the previous experiments in HAVSMCs. Tempol decreased intracellular ROS levels in HUVECs (Figure 5, (B1 and B3), *p* < 0.05), but Go 6983 did not decrease intracellular ROS levels.

### 3.10. Tempol and Go 6983 Decreased Intracellular Ca^2+^ Levels in HUVECs (Figure 5(c))

We detected intracellular Ca^2+^ levels in four groups of HUVECs after cold exposure though fluorescence microscopy and flow cytometry (Figure 5(c)). Cold exposure increased intracellular Ca^2+^ levels (*p* < 0.05), and Tempol reversed the increase in intracellular Ca^2+^ after cold exposure while Go 6983 did not (Figure 5, (C1–C4), *p* < 0.05).

### 3.11. Expression of ROS/PKC/ET-1 Pathway-Related Proteins in HUVECs (Figure 6)

**Figure 6:**
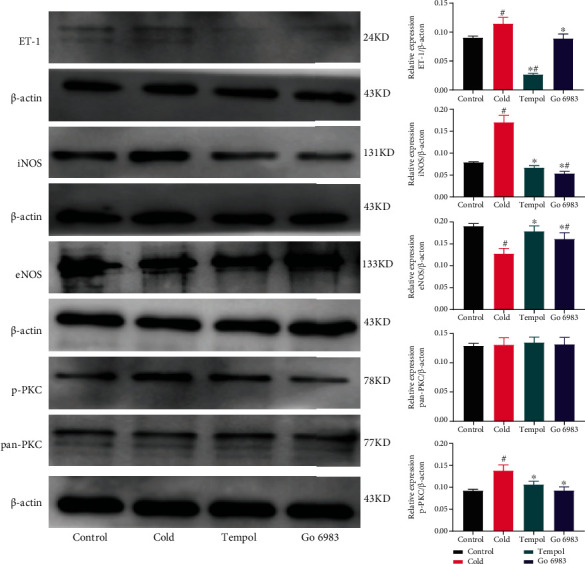
Expression of ROS/PKC/ET-1 pathway-related proteins in HUVECs. A cold environment increased the expression of ET-1, iNOS, and p-PKC (*p* < 0.05) and decreased eNOS levels (*p* < 0.05). Pretreatment with Tempol or Go 6983 decreased the expression of ET-1, iNOS, and p-PKC (*p* < 0.05) and increased the level of eNOS (*p* < 0.05). The experiments were repeated three times. ^∗^Compared with the cold group. ^#^Compared with the control group.

As shown in Figure 6, we found that a cold environment increased the expression of ET-1, iNOS, and p-PKC (*p* < 0.05), which resulted in lower eNOS levels (*p* < 0.05). Pretreatment with Tempol or Go 6983 decreased the expression of ET-1, iNOS, and p-PKC (*p* < 0.05) and increased the level of eNOS (*p* < 0.05). In addition, neither the environment nor drug treatment changed total PKC expression.

### 3.12. Signaling Pathway (Figure 7)

## 4. Discussion

This study provides evidence that ROS are vascular regulators that play a major role in cold-induced vasoconstriction. The development of a model of cooling that simulates cold exposure can provide new insight into the mechanisms underlying cold-induced vascular responses. The major findings are as follows: (1) cold exposure caused vasoconstriction, and ROS play a vital role in this process. (2) Reducing the level of vascular ROS instead of ROS in nerve fibers could inhibit vasoconstriction. (3) Cold exposure shrank HAVSMCs and HUVECs and made the cells narrower. (4) Cold increased ROS and Ca^2+^ levels in HAVSMCs and HUVECs, and an ROS neutralizer (Tempol) effectively inhibited intracellular ROS and reduced the level of intracellular Ca^2+^, while CCG-1423 and Go 6983 reduced the level of only intracellular Ca^2+^. (5) The signaling pathways involved in vasoconstriction include the ROS/RhoA/ROCK1 (HAVSMCs) and ROS/PKC/ET-1 (HUVECs) pathways.

Physiological research has described cold pain perception at low temperatures (<18°C) [14]. In this study, we utilized a new model of acute cold exposure [15] to quantitatively measure the vascular response after the hind limbs and buttocks of anesthetized mice had been cooled. Here, immediately after cold water immersion (4°C for 5 min), a dramatic decrease in blood flow was observed, followed by a gradual increase in blood flow. In addition, the same phenomena were observed in the mice.

ROS are a group of oxygen-derived molecules with one or more unpaired electrons in their outer orbital. Under normal conditions, ROS are essential signaling molecules that are tightly regulated to maintain physiological homeostasis and regulate cellular proliferation and host defenses. ROS can also participate in vasomotor responses, such as autoregulation, endothelium-dependent vasodilation, and flow-mediated vasodilation [14, 16]. In addition, ROS induce functional alterations in Ca^2+^ channels [17], which partly explains why cold exposure causes the Ca^2+^ concentration to increase. Tempol is a superoxide dismutase (SOD) analog that can effectively neutralize ROS. The injection of Tempol before cold exposure did not change systemic blood flow, emphasizing that a reduction in ROS in the normal mice could not regulate baseline blood flow. Previous studies have shown that a variety of antioxidants have beneficial therapeutic effects in animal models of pulmonary hypertension, supporting the role of ROS in the development of pulmonary hypertension [18, 19], which indicates that reducing oxidative stress can effectively suppress vasoconstriction. In this study, we found that reducing ROS levels in blood vessels but not nerves could effectively inhibit cold-induced vasoconstriction, similar to the results of previous studies.

**Figure 7:**
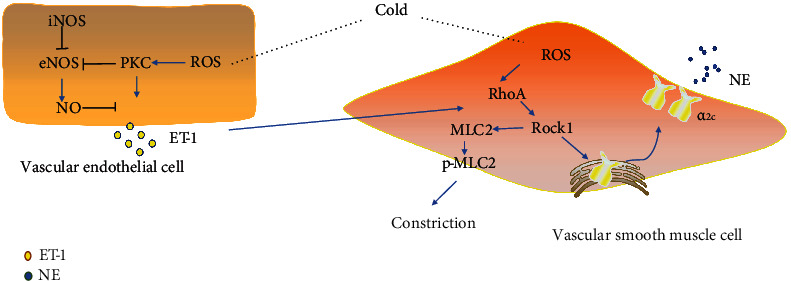
The mechanism of cold-induced vasoconstriction.

Superoxide has been suggested to induce vasoconstriction through the Rho kinase/ROCK pathway in VSMCs [20]. RhoA plays a primary role in regulating cellular contractility [21], and RhoA-induced contractility engages ROCK and further regulates VSMC mechanosensitivity in the microvasculature [22]. Chen et al. [23] found that ET-1 induced venular constriction in the porcine retina via activation of ROCK signaling. In this study, we found that elevated ROS could regulate the expressions of RhoA, ROCK1, and p-MLC-2, which indicated that activation of the ROS/RhoA/ROCK1 pathway can cause vasoconstriction (Figure 7). Elevated ROS levels did not affect the level of ROCK2 in HAVSMCs. Lee et al. [24] found that Danggui-Sayuk-Ga-Osuyu-Senggang-Tang-mediated activation of the RhoA/ROCK1/TESK1/PDXP pathway in cold-exposed pericytes appeared to be crucial for the treatment of vessel contraction. This is consistent with our research, which suggests that ROCK2 may not be involved in cold-induced vasoconstriction.

Cui et al. [25] found that TRAM34 can affect the biological functions of endothelial progenitor cells (EPCs) and accelerate cellular senescence through the NOX/ROS/PKC signaling axis. In HUVECs, cold induced increased ROS and p-PKC levels, while Tempol reduced p-PKC levels, and Go 6983 did not reduce intracellular ROS levels, indicating that ROS are upstream of PKC and capable of modulating the phosphorylation of PKC (Figure 7). NO, which promotes vasodilation, is constitutively produced by eNOS. The crossmodulation of eNOS and iNOS activity in the cardiovascular system is a crucial event [26] that regulates the release of NO to maintain vasodilation. In this study, we found that ROS could decrease the eNOS level and increase iNOS expression, which inhibited the synthesis and release of NO, leading to vasoconstriction. Pretreatment with Tempol or Go 6983 reversed these two changes (Figure 7).

ET-1 derived from endothelial cells is the most potent vasoconstrictor in the human cardiovascular system and has remarkably long-lasting effects [27]. Cold exposure not only directly triggered an increase in plasma ET-1 secretion but also caused VSMC contraction, vasospasm, emboli, and even local tissue ischemia and edema [28]. In this study, we found that cold exposure-induced vasoconstriction and cold exposure led to elevated ET-1 levels in HUVECs, which confirmed the results of previous studies.

## 5. Conclusion

A cold environment can induce skin vasoconstriction and reduce blood flow. This study shows that ROS in blood vessels rather than nerve fibers play an important role in vasoconstriction induced by cold exposure via the ROS/RhoA/ROCK1 and ROS/PKC/ET-1 pathways in VSMCs and endothelial cells.

## Figures and Tables

**Figure 4:**
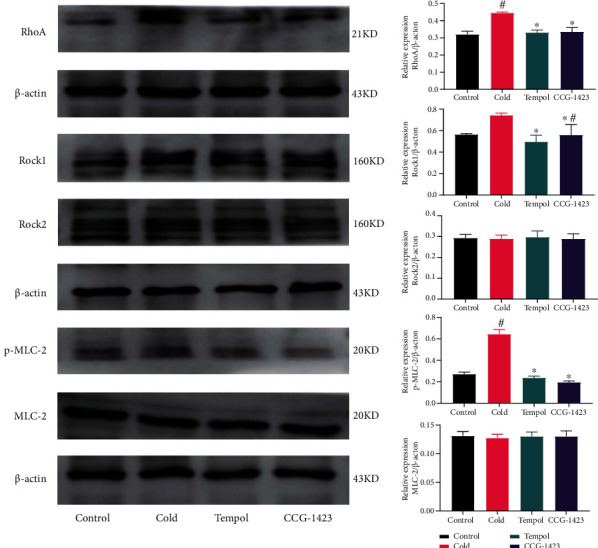
Expression of ROS/RhoA/ROCK1 pathway-related proteins in HAVSMCs. Cold increased the expression of RhoA, Rock1, and p-MLC-2 compared to those in the other groups (*p* < 0.05). Tempol and CCG-1423 decreased the levels of RhoA, Rock1, and p-MLC-2 (*p* < 0.05). The experiments were repeated three times. ^∗^Compared with the cold group. ^#^Compared with the control group.

## Data Availability

The data used to support the findings of this study are available from the corresponding author upon request.

## Abbreviations

eNOS: Endothelial nitric oxide synthase
ET-1: Endothelin-1
HAVSMC: Human aortic smooth muscle cell
HUVEC: Human umbilical vein endothelial cell
iNOS: Inducible nitric oxide synthase
ICR: Institute of Cancer Research
MLC-2: Myosin light chain
PKC: Protein kinase C
RhoA: Ras homolog gene family, member A
ROS: Reactive oxygen species.
